# Global inference of disease-causing single nucleotide variants from exome sequencing data

**DOI:** 10.1186/s12859-016-1325-x

**Published:** 2016-12-23

**Authors:** Mengmeng Wu, Ting Chen, Rui Jiang

**Affiliations:** 1 MOE Key Laboratory of Bioinformatics; Bioinformatics Division and Center for Synthetic & Systems Biology, Tsinghua National Laboratory for Information Science and Technology, Beijing, China; 2 Department of Computer Science, Tsinghua University, Beijing, China; 3 Department of Automation, Tsinghua University, Beijing, China

## Abstract

**Background:**

Whole exome sequencing (WES) has recently emerged as an effective approach for identifying genetic variants underlying human diseases. However, considerable time and labour is needed for careful investigation of candidate variants. Although filtration based on population frequencies and functional prediction scores could effectively remove common and neutral variants, hundreds or even thousands of rare deleterious variants still remain. In addition, current WES platforms also provide variant information in flanking noncoding regions, such as promoters, introns and splice sites. Despite of being recognized to harbour causal variants, these regions are usually ignored by current analysis pipelines.

**Results:**

We present a novel computational method, called Glints, to overcome the above limitations. Glints is capable of identifying disease-causing SNVs in both coding and flanking noncoding regions from exome sequencing data. The principle behind Glints is that disease-causing variants should manifest their effect at both variant and gene levels. Specifically, Glints integrates 14 types of functional scores, including predictions for both coding and noncoding variants, and 9 types of association scores, which help identifying disease relevant genes. We conducted a large-scale simulation studies based on 1000 Genomes Project data and demonstrated the effectiveness of our method in both coding and flanking noncoding regions. We also applied Glints in two real exome sequencing and demonstrated its effectiveness for uncovering disease-causing SNVs. Both standalone software and web server are available at our website http://bioinfo.au.tsinghua.edu.cn/jianglab/glints.

**Conclusions:**

Glints is effective for uncovering disease-causing SNVs in coding and flanking noncoding regions, which is supported by both simulation and real case studies. Glints is expected to be a useful tool for human genetics research based on exome sequencing data.

**Electronic supplementary material:**

The online version of this article (doi:10.1186/s12859-016-1325-x) contains supplementary material, which is available to authorized users.

## Background

Technical advancement in whole exome sequencing (WES) has enabled the rapid and cost-efficient detection of variants in exonic regions or nearby, promoting the identification of causative variants underlying Mendelian diseases [1], complex disorders [2], and cancers [3]. Nevertheless, computational analysis of WES data still remains a great challenge, due to the fact that the number of distinct variants in a study usually increases dramatically with the increase of the size of a disease cohort, and a significant proportion of these variants occur in extremely low frequency [4]. In addition, WES can also discover a non-negligible fraction of variants occurring in flanking regions of captured exons [5, 6]. These regions, with examples including promoters, splice sites and introns, though having been recognized to harbor causal variants [7], are typically ignored by current analysis pipelines.

Confronting the first challenge, two groups of computational methods have been developed for prioritizing candidate variants from WES data. Specifically, targeting on nonsynonymous single nucleotide variants (nsSNVs), the first group of methods, with such representative examples as SIFT [8] and PolyPhen2 [9], utilize sequence, biochemical and evolutionary information of amino acids to predict functionally damaging effects of variants. Some methods, like Condel [10], are developed to integrate multiple functional predictions to provide more accurate predictions. However, such prediction scores, though having been announced with high accuracy in such public data sets as HGMD [11], Siwss-prot [12] and ClinVar [13], usually have high false positives and low explanatory power in real experimental studies [14, 15]. To overcome this limitation, the second group of methods, represented by eXtasy [16], SPRING [17] and snvForest [18], integrate multiple functional predictions of variants, association information between genes and diseases, as well as phenotype information to prioritize candidate variants. There also exist several methods integrating variant functional predictions and disease-gene association to prioritize disease genes, such as PHIVE [19] and Phen-Gen [20]. The difference between variant prioritization and gene prioritization is significant as former incorporates disease-gene association into variants while latter aggregates variant functions into genes. However, these methods, though capable of eliminating false positives, usually rely heavily on prior knowledge about the disease under investigation to make inference. For example, SPRING takes a set of seed genes known as associated with the disease of interest as input. In the case that a query disease has never been investigated for genetic basis, genes associated with 10 diseases of the highest phenotype similarities with the query disease are used as seeds. This strategy, though proved to be valid, can hardly be optimal, since the association information between genes and other diseases are all ignored. In other words, this strategy has the local property because only diseases having very high phenotype similarity with the query disease contribute to the inference procedure.

As for the second challenge, the prediction of functionally damaging effects of noncoding variants is much more difficult than coding variants. Unlike variants in coding region, noncoding variants affect biological functions through such complex mechanisms as epigenetic regulation [21]. Fortunately, with the recent development in epigenomics and the release of such large-scale projects as ENCODE [22] and Roadmap Epigenomics [23] that aim at dissecting regulatory elements, the prediction of functional effects of noncoding variants has now become feasible, leading to such methods as CADD [24], FunSeq [25], GWAVA [26], DeepSEA [27], deltaSVM [28]. Nevertheless, to the best of our knowledge, there still lacks a computational method capable of predicting causative noncoding variants for a specific type of disease.

To overcome the above limitations, we propose a novel computational method, called Glints, to prioritize both coding and flanking noncoding variants in a disease-specific manner by integrating 14 types of functional scores and 9 types of association scores. We extracted functional scores for SNVs from dbWGFP [29], a repository collecting whole genome SNVs and their functional predictions, and devised a multivariate regression model to quantify association scores between candidate genes and diseases of interest. After converting both of functional scores and association scores into *p*-values, we integrated them with Fisher’s combined probability test. We conducted a large-scale simulation studies based on 1000 Genomes Project Phase I data and demonstrated the effectiveness of our method for identifying causal variants in both coding and flanking noncoding regions. We further compared our method with several existing methods for prioritizing coding nsSNV, and demonstrated the superior performance of our method. We applied our method to two real exome sequencing data, and found that Glints could uncover known causal variants and discover new variants with high causality probabilities. Thus, Glints is expected to contribute to human genetics studies based on exome sequencing, and facilitates our understanding about human diseases.

## Results

### Overview of Glints

**Fig. 1:**
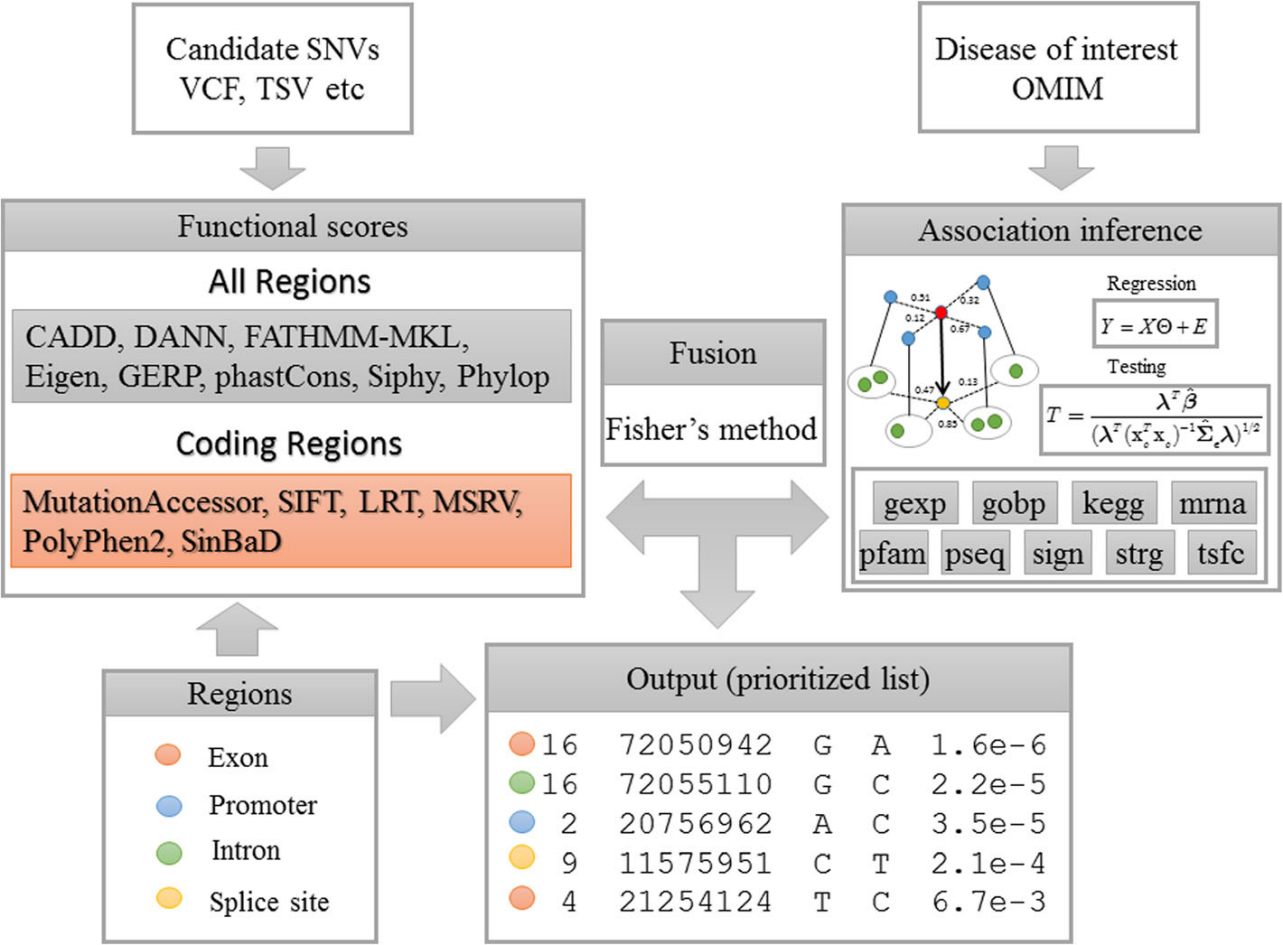
Schematic overview of Glints. Glints requires input candidate SNVs (e.g. VCF) and query disease of interest. The process of Glints consists of four parts: 1) annotate each SNV into four regions as Exon, Promoter, Intron and Splice site; 2) select and extract functional scores for each candidate SNV according to its region; 3) infer association between genes hosting candidate SNVs and query disease via multivariate regression; 4) integrate both variant-level and gene-level information via Fisher’s method and produces statistical significance (*q-value*) for each SNV

The workflow of Glints is illustrated in Fig. 1. Taking a list of candidate single nucleotide variants (SNVs) and a query disease as input, Glints takes four steps to calculate predictive scores for variants and produce a ranking list that prioritizes SNVs according to their potential for causing the query disease. The first step is to categorize candidate SNVs into different groups according to their relative positions to genes and possible effects on protein functions. With the help of bioinformatics tools like ANNOVAR [30], we classify candidate SNVs into four subgroups: 1) Exon, 2) Promoter, 3) Intron and 4) Splice site. Specifically, Exon refers to nonsynonymous SNV in coding region, Promoter refers to regions overlapping 500 bp upstream of TSS plus UTR5 and UTR3 regions, Intron refers to inner 3–10 bp regions from exon/intron boundaries, and Splice site refers to inner 2 bp regions from exon/intron boundaries (also called canonical splice site). SNVs located at other regions, e.g. intergenic, are discarded. In the second step, we annotate each variant with functional prediction scores of its functionally damaging effect according to its group information. Specifically, we select 8 types of whole-genome scores, named CADD [24], DANN [31], FATHMM-MKL [32], Eigen [33], GERP [34], phastCons [35], Siphy [36], Phylop [37], for all these four regions and another 6 types of protein function scores, named MutationAccessor [38], SIFT [8], LRT [39], MSRV [40], PolyPhen2 [9] and SinBaD [41], for coding regions only. We convert these functional scores to *p*-values for subsequent integration. In the third step, we identify genes hosting these SNVs and derive 9 association scores to characterize the potential association between the genes and the query disease. This is done by resorting to a multivariate regression model that explains 3 types of disease phenotype similarities (i.e., UMLS [42], MeSH [43] and HPO [44], detailed in Additional file 1: Section 1) by using one type of gene functional similarity measure (i.e., gene expression [45], gene ontology [46], KEGG pathway [47], microRNA regulation [48], protein domain [49], protein sequence [50], signaling pathway [51], protein-protein interaction [51], and transcriptional regulation [52], detailed in Additional file 1: Section 2). We further convert the resulting association scores to *p*-values for subsequent integration. Finally, we apply the Fisher’s method [53] to integrate the calculated *p*-values at both the variant and gene levels with the consideration of dependence correlations between the data sources, and we perform multiple testing correction by calculating *q*-values from integrated *p*-values according to a statistical method called pFDR [54]. The final *q*-values provide a means for prioritizing candidate SNVs.

### Contributions of our method

Compared with other methods, including our previous work, Glints makes three main contributions: 1) Glints introduces a multivariate regression method for inferring disease-gene association, in which three types of phenotypic similarities (UMLS, HPO, MeSH) are integrated. In contrast, our previous work pgFusion [55] utilized only one type of phenotypic similarity (UMLS) with a univariate regression method. 2) Glints incorporates disease-gene association obtained by multivariate regression into variant prioritization, and utilize global network information. Previous work, such as eXtasy [16], SPRING [17], PHIVE [19] and snvForest [18] only utilize disease-gene information locally, such as gene associated several similar diseases, also called “seed genes”. Thus, these methods could be ineffective on some diseases whose similar diseases have no associated genes or “seed genes” are not available. 3) Glints incorporates variant functional predictions and disease-gene association to prioritize flanking noncoding variants in disease-specific manner, which remains unexplored to the best of our knowledge.

### Simulation studies

We validated our method using large-scale simulation data based on the 1000 Genomes Project Phase I data for both coding and flanking noncoding regions. For this purpose, we collected known causal variants from HGMD, which is the largest repository for collecting disease-causing variants, including coding, regulatory and splicing variants. Since our inference of association scores is based on OMIM, we performed mapping between HGMD disease descriptive texts and OMIM identifiers. We regarded it as matched if one of the following criterions was satisfied: 1) HGMD description exactly matched OMIM description; 2) HGMD disease and OMIM disease shared the same causal variants, either in DNA sequence format or rsid; 3) HGMD disease and OMIM disease shared the same pubmed ID. Obviously, such rules could introduce inexact mappings, for example, those variants showing pleiotropic effects could link different diseases. Thus, when associating a variant from HGMD with OMIM, we selected the OMIM uniquely mapped to the HGMD text description of the variant and discarded those variants which were mapped into multiple OMIMs. With such strict filtering, we discarded many candidate variants in order to ensure the quality of the remaining variants. Finally we compiled a dataset consisting of 9872 causal variants (shown in Table 1) with high reliability in both coding (exon) and flanking noncoding regions (promoter, intron and splice site) and used them as ground truth. For each of the four regions, we extracted corresponding SNVs from the 1000 Genomes Project and used them as controls. For example, we extracted all SNVs in promoter regions of each individual from 1000 Genomes Project and these variants were used as controls for promoter regions. Different functional scores varied significantly in terms of coverage across different regions, as shown in Additional file 1: Table S1, highlighting the advantage of data integration as improving coverage. Some of these functional predictions, such as CADD, DANN, Eigen etc, utilize neutral variants from 1000 Genomes Project to build predictive model (Additional file 1: Table S2), which may result in circulatory validation and overestimated results. Thus, we also assessed performance of Glints after excluding these functional predictions (Table 2).

**Table 1:** Summary statistics for data used in simulated experiment across different regions

		Exon	Promoter	Intron	Splice site
Causal	Variant	8350	114	303	1105
	Gene	1063	34	132	280
Control (average)	Variant	9512	18,181	2532	78
	Gene	5336	8486	2102	77

For control, the numbers of neutral variants across different regions are average number of corresponding neutral variants in 1092 individuals from the 1000 Genomes Project Phase I

**Table 2:** The prioritization performance of Glints and individual scores on 1000 Genomes Project based simulated data

Method	Exon			Promoter			Intron			Splice site		
	TOP	MRR	AUC	TOP	MRR	AUC	TOP	MRR	AUC	TOP	MRR	AUC
CADD	171	12.86%	87.13%	0	14.66%	85.33%	7	20.29%	79.71%	776	13.33%	87.14%
DANN	108	10.97%	89.03%	0	18.99%	81.02%	35	18.60%	81.41%	844	9.21%	91.29%
FATHMM-MKL	127	11.80%	88.19%	0	15.20%	84.80%	74	11.25%	88.70%	850	8.67%	91.84%
Eigen	95	5.47%	94.50%	29	21.97%	78.05%	4	14.95%	85.06%	218	19.89%	80.52%
LRT	0	13.17%	86.95%	NA	NA	NA	NA	NA	NA	NA	NA	NA
MSRV	1872	7.53%	92.38%	NA	NA	NA	NA	NA	NA	NA	NA	NA
MutationAccessor	583	9.81%	90.16%	NA	NA	NA	NA	NA	NA	NA	NA	NA
PolyPhen2	0	8.25%	91.77%	NA	NA	NA	NA	NA	NA	NA	NA	NA
SinBaD	150	7.62%	92.36%	NA	NA	NA	NA	NA	NA	NA	NA	NA
SIFT	0	13.64%	86.29%	NA	NA	NA	NA	NA	NA	NA	NA	NA
GERP	58	16.49%	83.51%	2	24.29%	75.70%	50	17.01%	82.96%	736	12.43%	88.06%
Siphy	60	36.37%	63.63%	8	23.70%	76.30%	5	47.26%	52.75%	391	35.16%	65.06%
Phylop	119	12.96%	87.02%	19	26.24%	73.73%	79	15.87%	84.06%	863	9.03%	91.46%
PhastCons	0	14.12%	85.78%	0	28.52%	71.48%	0	15.88%	84.06%	585	16.22%	84.21%
gexp	1330	17.36%	82.61%	55	21.18%	78.77%	101	21.25%	78.74%	673	20.59%	79.80%
gobp	3006	10.44%	89.40%	56	11.47%	88.33%	173	10.09%	89.78%	937	11.03%	89.39%
kegg	2321	20.21%	79.85%	10	19.53%	80.44%	143	20.30%	79.77%	737	22.16%	78.34%
mrna	1462	24.25%	75.73%	31	30.62%	69.52%	96	32.99%	67.05%	437	29.47%	70.86%
pfam	2297	17.69%	82.30%	53	20.93%	79.13%	137	20.49%	79.54%	717	20.02%	80.42%
pseq	1194	22.21%	77.87%	7	25.21%	74.91%	55	23.15%	76.96%	697	23.78%	76.70%
sign	1447	28.10%	72.07%	54	25.19%	74.91%	111	32.49%	67.75%	507	31.01%	69.49%
strg	3086	10.96%	88.92%	57	6.31%	93.46%	140	11.51%	88.39%	922	11.28%	89.17%
tsfc	1248	30.07%	69.83%	37	35.64%	64.28%	103	33.45%	66.47%	490	33.58%	66.59%
Glints^a^	4646	2.12%	97.61%	82	4.51%	95.26%	209	4.12%	95.68%	1012	5.20%	95.29%
Glints	4736	2.12%	97.62%	82	3.63%	96.20%	219	3.65%	96.13%	1047	4.06%	96.43%

NA denotes unavailability of the individual score on corresponding region. Glint^a^ denotes conservative results of Glints after excluding CADD, DANN, FATHMM-MKL, MSRV and SinBaD. TOP denotes number of causal variants ranked in top 10, MRR denotes mean rank ratio and AUC denotes area under rank ROC. Some abbreviations for score name: *gexp* gene expression, *gobp* gene ontology, *kegg* KEGG pathway, *mrna* microRNA regulation, *pfam* protein families, *pseq* protein sequence, *sign* signaling pathway, *strg* protein-protein interaction, *tsfc* transcriptional regulation

For each of the four different regions, we separately spiked each causal variant from the region into the pool of corresponding control SNVs of each individual from 1000 Genomes Project to simulate real sequencing data. We then prioritized the mixed SNVs using Glints and observed the relative positions of causal SNVs in the final ranking list. In order to eliminate bias and possible information leakage, we removed all known genes associated with the query disease to mimic the scenario under which the genetic basis for the query disease was totally unknown. To evaluate the performance of our method quantitatively, we defined the rank ratio of a test variant as the rank of the variant divided by the number of neutral variants and the mean rank ratio (MRR) of a disease as the average rank ratio of causal variants corresponding to that disease. We then took the average MRR for these diseases as a metric to assess overall performance. Since a smaller MRR means that disease-causing variants are enriched in the top-ranking positions, better performance is indicated with smaller MRR. We could also obtain both false-positive and true-positive rates by defining a threshold for rank ratio and easily compute the area under ROC curve (AUC) through varying the threshold. Another important indicator is the number of causal variants which are ranked in the top 10 (TOP), and a higher TOP number means higher performance.

**Fig. 2:**
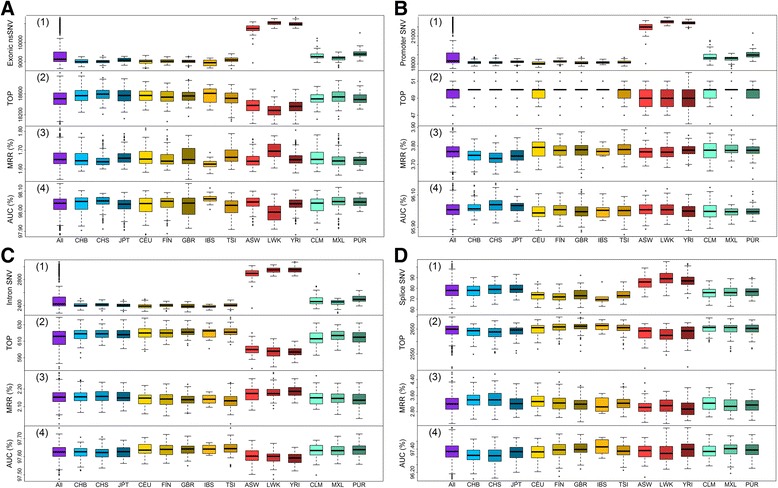
Results on simulation studies based on sequencing data from 1000 Genome Project. For each region, results on simulation studies are summarized as four metrics: (1) the number of neutral variants; (2) TOP, the number of causal variants ranked in top 10; (3) MRR, the average rank ratio of causal variants; (4) AUC, the average area under the rank ROC. Regions are categorized into **a** Exon; **b** Promoter; **c** Intron; **d** Splice site. The x axis denotes different populations. Population abbreviations: ASW, people with African ancestry in Southwest United States; CEU, Utah residents with ancestry from Northern and Western Europe; CHB, Han Chinese in Beijing, China; CHS, Han Chinese South, China; CLM, Colombiansin Medellin, Colombia; FIN, Finnish in Finland; GBR, British from England and Scotland, UK; IBS, Iberian populations in Spain; LWK, Luhya in Webuye, Kenya; JPT, Japanese in Tokyo, Japan; MXL, people with Mexican ancestry in Los Angeles, California; PUR, Puerto Ricans in Puerto Rico; TSI, Toscani in Italia; YRI, Yoruba in Ibadan, Nigeria. Ancestry-based groups: AFR, African; AMR, Americas; EAS, East Asian; EUR, European

We first evaluated the ability of Glints to prioritize nsSNVs (Fig. 2(a)). The average number of nsSNVs in each exome was around 9000 ~ 10,000. The three African populations had a slightly higher number of nsSNVs, which is consistent with previous findings. The average number of causal variants ranked in the top 10 was around 4736, out of 8350, demonstrating that more than one half of causal nsSNVs can be prioritized in top ranks by our method. We also noticed that the three African populations had a slightly smaller number of top 10-ranked variants with average 4686 in top 10, consistent with the fact that these populations have relatively higher number of neutral variants. The overall average MRR was around 2.12%, and the corresponding AUCs were around 97.6%. We found significant difference on performance across different populations with ANOVA analysis and p-values were 3.24 × 10^-12^, 5.42 × 10^-16^ and 2.06 × 10^-14^ for Top, MRR and AUC. The differences can be attributed to difference on number of candidate variants across different populations (*p-values* < 2 × 10^-16^for all these four regions) and significant relationship between performance and candidate number (Additional file 1: Figure S1).

We then evaluated the ability of Glints to prioritize promoter SNVs. To accomplish this, we performed the 1000 Genomes Project-based simulation studies, as noted above. Since each individual harbors 19,055 Promoter SNVs on average (Fig. 2(b)), pinpointing causal Promoter SNVs from this large pool is challenging. Nonetheless, Glints achieved a satisfactory result with about 82 of 114 causal promoter SNVs ranked in the top 10, significantly more than expected by chance. The average MRR is 3.63%, and corresponding AUCs is 96.20%, both better than expected by chance. We also observed significant difference on performance across different populations (*p-values* < 2 × 10^-16^ for Top, MRR and AUC), but this difference cannot be attributed to varying size of candidate number across different populations except for Top (Additional file 1: Figure S1).

We next evaluated the ability of Glints to prioritize intronic SNVs by the same method described above. As shown in Fig. 2(c), on average, each individual carries about 2532 intronic variants when considering inner 3-10 bp only, with the single exception of these three African populations, who carry more (*p-value* < 2 × 10^-16^). Glints ranked about 219 out of 303 causal variants in the top 10, which was significantly better than expected by chance. The corresponding MRR and AUC were 3.65 and 96.13%, respectively, on average, suggesting the effectiveness of our method.

Finally, Glints was evaluated for its ability to prioritize splice site SNVs, still using the same method as described above. As shown in Fig. 2(d), on average, each individual has 80 splice site variants. Glints ranked 1047 out of 1105 causal splice site variants in the top 10. We observed a greater proportion of splice site variants receiving top ranking as a result of the smaller number of candidates in this region. The corresponding MRR and AUC were 4.06 and 96.43%, respectively, on average, again suggesting the effectiveness of our method.

### Comparison with existing methods

**Fig. 3:**
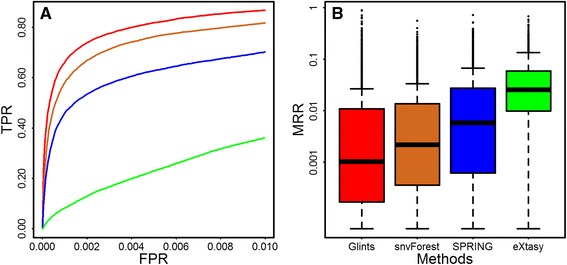
Comparsion of Glints with existing methods on prioritization of nsSNV. Comparsion is performed on disease variants and neutral variants from Swiss-prot database. Both partial rank ROC (**a**) and boxplot (**b**) indicate the superior performance of Glints

To the best of our knowledge, Glints is the first method able to prioritize flanking noncoding variants in a disease-specific manner. Therefore, we only performed comparison between our method and existing approaches on nsSNVs. We selected three representative methods, termed eXtasy [16], SPRING [17] and snvForest [18], and compared them with Glints. We excluded PHIVE [19] and Phen-Gen [20] from comparison since they were designed to prioritize candidate genes and we found them unsuitable for variant prioritization via the same simulation studies. From the Swiss-Prot database, we collected 24,300 disease variants and 38,910 neutral variants for evaluation. To maintain consistency with the original studies of SPRING, we sampled half of the neutral variants for testing. Each disease variant was ranked against 19,455 neutral variants, and performance was evaluated using MRR and AUC, as defined above. We set a rank ratio threshold and calculated the false-positive rate as the fraction of neutral variants whose rank ratios were below the threshold and true-positive rate as the fraction of disease variants whose rank ratios were below the threshold. By varying the threshold from 0 to 1, we drew a curve similar to ROC and called it rank ROC, which is used for gene prioritization [56]. From Fig. 3(a), we observed obvious advantages of Glints over the other three methods. For example, at false-positive rate of 1%, true-positive rates are 86.8, 81.7, 70.2 and 36.1% for Glints, snvForest, SPRING and eXtasy respectively. Here, we focused on the performance with false positive rate below 1% to evaluate the performance of these methods on discovering real causal variants while controlling for false positive rate meanwhile. For the 1478 diseases tested, we took the mean rank ratio of corresponding causal variants as the MRR of diseases and drew their distribution in Fig. 3(b), which also showed that Glints outperformed other methods. Specifically, the mean MRR of these 1478 diseases for Glints, snvForest, SPRING and eXtasy were 0.10, 0.22, 0.59 and 2.56% respectively. We also used one-sided Wilcoxon test to assess significance of difference and found that Glints gave significant lower MRRs than the other three methods. The *p*-values for comparing Glints with snvForest, SPRING and eXtasy are 4.31 × 10^-8^, 6.77 × 10^-33^ and 8.44 × 10^—172^ respectively. We also evaluated the performance of these three methods on 1000 Genomes based simulation studies as above and found that Glints assigned significant topper ranks to causal variants with *p*-values as 2.8 × 10^-69^, 0 and 0 for snvForest, SPRING and eXtasy respectively. These results collectively suggest superior performance of Glints over alternative methods.

### Contribution of individual scores

We included 14 types of different functional prediction scores in Glints, and these scores differed in several aspects, such as principles, training data, learning algorithms and applicability etc (Additional file 1: Table S2). Even for the same score, difference may exist when applied in different regions. We first evaluated the correlations between these functional prediction scores and scores with similar underlying learning procedures are expected to show high correlations and tend to cluster together. For each of these four regions, we selected corresponding causal variants used in aforementioned simulation studies and corresponding functional scores. We then computed Pearson’s correlation coefficients between each pair of functional scores. SIFT and LRT were transformed with 1-SIFT and 1-LRT respectively, in order to keep consistency in direction for expressing deleteriousness, e.g. higher values indicating higher deleteriousness.

**Fig. 4:**
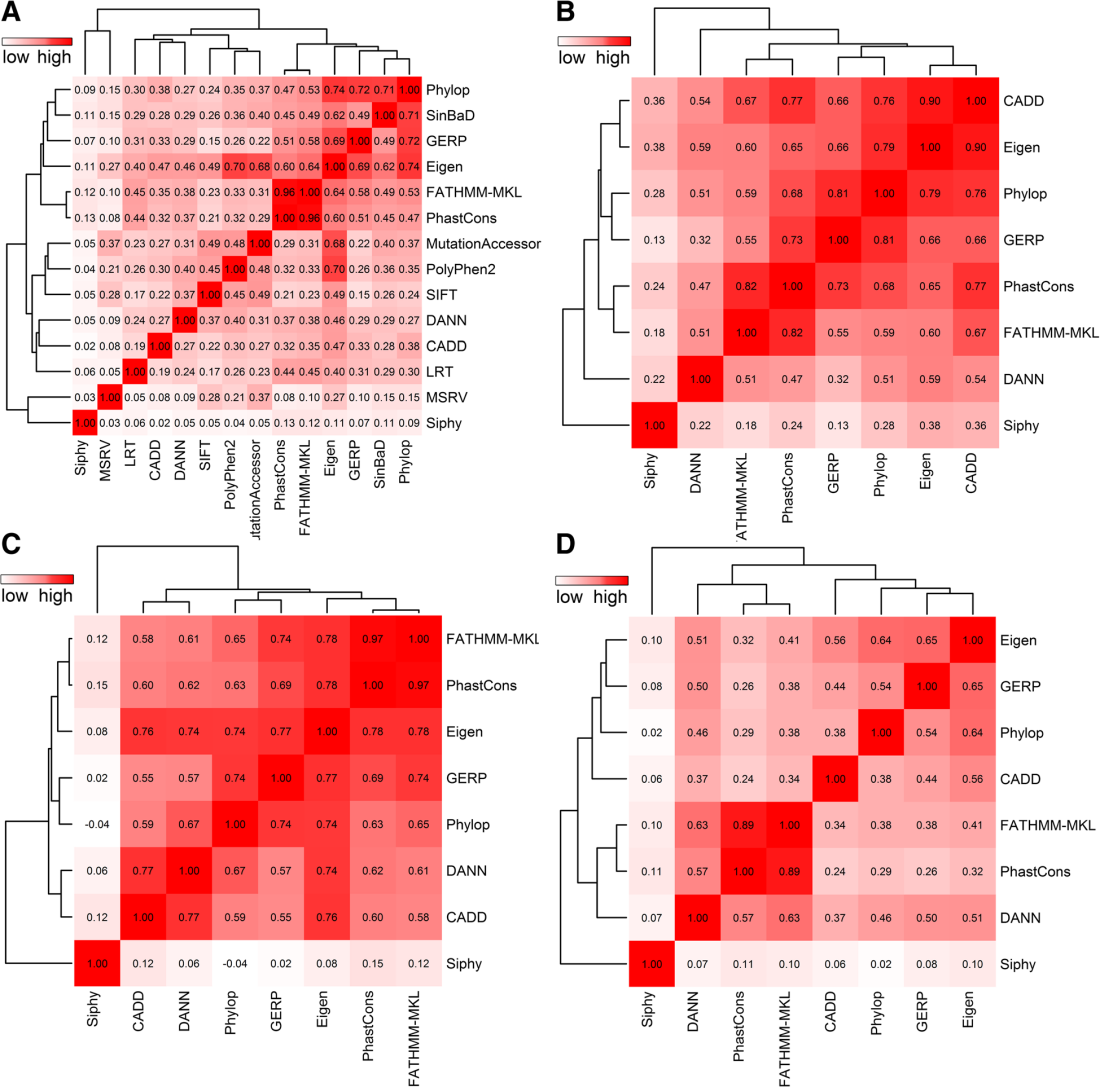
Correlations between functional prediction scores across different regions. Pearson’s correlation coefficients are calculated from causal variants across different regions: **a** Exon, **b** Promoter, **c** Intron, **d** Splice site

As shown in Fig. 4, we observed different patterns in these regions. The correlations between PhastCons and FATHMM-MKL were 0.96, 0.82, 0.97 and 0.89, for exonic, promoter, intron and splice site respectively, which were consistent and high across all regions, and they clustered together all the time, possibly because both methods relied on multiple genome alignment and FATHMM-MKL put high weight on evolutionary information via multiple kernel learning. Apart from PhastCons and FATHMM-MKL, some obvious, but different, correlations existed in those regions. For the exonic region, we observed obvious correlations between Phylop, SinBaD, GERP and Eigen with possible reason that the former three methods rely heavily on evolutionary conservation and Eigen incorporates such information with high weight via unsupervised learning. In addition, Eigen also has high correlations with PolyPhen2 and MutationAccessor, due to these two scores are also incorporated by Eigen. For the promoter region, an obvious correlation existed between Phylop, GERP and PhastCons, as well as between CADD and Eigen. For the intronic region, we observed correlations between all methods except Siphy. Although Siphy relied on evolutionary conservation as the others did, it used multiple sequence alignment of ENOCDE regions as training data while the others used multiple sequence alignment of different species genome. For the splice site region, we observed correlation between Eigen and GERP. We also observed that SiPhy had almost no correlations with other methods. Additionally, the correlation between CADD and DANN was low, except for the intronic region. Although both methods use the same training data, they differed in that CADD used linear SVM, while DANN used deep neural network. Thus, DANN is able to discover more nonlinear relationships than SVM, and, hence, it exhibits better performance on several testing datasets [57]. We also found that DANN outperformed CADD in the exonic, intron and splice site regions when tested individually (as shown in Table 2), while CADD had better performance in the promoter region. Thus, DANN and CADD supplied different information, making it essential to include both of them in our method.

We assessed the performance of each individual score in each region by repeating the same simulation studies with the score only, as shown in Table 2. For example, several scores were only available for exonic nsSNVs, including LRT, MSRV, SIFT, SinBaD, MutationAccessor and PolyPhen2. Therefore, we did not assess their performance in regions other than exon. All gene scores were not restricted to single variant, thus were available for all regions. From Table 2, we clearly saw the advantages of integrating multiple data sources, which resulted in better performance when compared to all individual scores. For example, in the exonic region, MRRs of individual scores ranged from 5.47 to 36.37%, while corresponding TOPs range from 0 to 3086. In contrast, with integration, Glints achieved an MRR of 2.12% and TOP of 4736. In the promoter region, MRRs of individual scores range from 6.31 to 30.62%, while corresponding TOPs range from 0 to 57. With integration, Glints achieved an MRR of 3.63% and TOP of 82. In the intronic region, MRRs of individual scores ranged from 10.09 to 47.26%, while corresponding TOPs ranged from 0 to 143. With integration, Glints achieved an MRR of 3.65% and a TOP of 219. Finally, in the splice site region, MRRs of individual scores ranged from 9.03 to 35.16%, while corresponding TOPs ranged from 218 to 922. With integration, Glints achieved an MRR of 4.06% and TOP of 1047. We also removed CADD, DANN, FATHMM-MKL and Eigen from Glints due to their usage of 1000 Genomes Project as training data, and the resulting Glints also show better performance than any individual score (Table 2).

### Application on real sequencing data

In order to assess the effectiveness of Glints on real sequencing data, we collected two recently published exome sequencing data and applied Glints to them. We only assessed Glints’ performance on coding variants due to difficulty of accessing data for flanking noncoding variants. The first case was a study on epileptic encephalopathies (MIM: 615369), which described a heterogeneous and deleterious group of childhood epilepsy disorders with syndromes associated with severe cognitive and behavioral disturbances. In this study [58], 264 probands with their parents were recruited for exome sequencing, and strong statistical evidence on the association between *de novo* mutations with this disorder was found. In total, we collected 192 candidate nonsynonymous *de novo* mutations from this study and applied Glints to prioritize them with the objective of identifying functional mutations. Of those candidates, 30 mutations were reported to show obvious statistical evidence in the original literature; therefore, those mutations were considered functional. Using the identical procedure as that used in the simulation experiments described above, we removed all known genes associated with this disorder and all genes overlapping those candidate mutations to prevent possible information leakage. In the prioritized list, 23 out of the top 25 were functional, highlighting the capability of our method for this case. A one-sided Fisher’s exact test suggests that the probability of ranking 23 functional mutations among the top 25 by chance is only 2.14 × 10^-14^, further supporting our method for enriching functional mutations in top positions. Among those top 25 mutations, two mutations were not reported as functional in the original study. One was on gene GABRB1, which was recently proved to be associated with thalamus volume and intelligence [59]. The other one was on gene GNAO1, which was also recently reported to play a significant role in epileptic encephalopathy [60, 61]. In comparison, SPRING ranked 17 functional SNVs among top 25, and the numbers for eXtasy and snvForest were both also 17.

Another case was a study on autism spectrum disorders (ASD, MIM: 209850) and Neale et al. [62] sequenced the exomes of 175 ASD cases and their parents. Through statistical modeling of *de novo* mutations in the cohort, several genes were identified as key factors involved in ASD with strong evidence. From this study, we collected a total of 104 synonymous SNVs as candidates, among which five were reported as likely functional in the original study. We applied Glints to this list of candidates using a strategy similar to that described above in order to eliminate possible information leakage. In the final prioritized list, five functional SNVs received ranks of 1, 2, 9, 11 and 38. A one-sided Fisher’s exact test suggests that the probability of ranking 3 functional SNVs in the top 10 by chance is only 0.022. In comparison, SPRING gave these functional variants ranks of 2, 3, 7, 11, 52, while eXtasy gave ranks of 5, 8, 17 and filtered out two functional variants. snvForest gaves ranks of 4,9,10,11,70 to these functional variants. These two real cases both indicated better performance of Glints than the other three methods on real exome sequencing data.

## Discussion

It is also worth noting that several aspects of our method can be improved in the future. First, our method is restricted to flanking noncoding regions that are nearby gene regions. Intergenic regions are not suitable for the application of our method since it is hard to assign gene to variations that locate at these regions. It is technically feasible to apply our method to deeper intronic regions, but we cannot evaluate its performance without available data for this region. With the accumulation of variation data and advance in assigning genes to intergenic variants, our method can be extended to handle these regions. Second, our method is restricted to single nucleotide variants, but several other kinds of variants, such as indel, structural variation etc, are also important for human diseases. How to extend our methodology to other forms of variations is one research direction for future. In addition, our method for integration can also inspire methodological developments for integration of other types of biological data. The volume of genomic and genetic data has increasingly accumulated, but how to integrate such bulky data to distill meaningful biological insights is far from trivial. Analogy to our method, each type of genomic data can be converted into *p*-values followed by integration with weighted Fisher’s method. The combined p-values represent collective evidence from a variety of data, and can effectively reduce false positives compared with single type of data.

One major challenge for developing computational methods for identifying causative variants is the scarcity of public real sequencing data. After surveying hundreds of literatures, we find only two exome sequencing data and real data for noncoding variants is not available. In the future, increasing number of public sequencing datasets will benefit the methodological development.

## Conclusions

In this study, we present a novel computational method, called Glints, to prioritize both coding and flanking noncoding SNV with respect to the query disease in exome sequencing studies. It can also be useful in whole sequencing studies if only coding and flanking noncoding variants are focused. Our method integrates 14 types of functional prediction scores for variants, including predictions for both coding and noncoding regions, and 9 types of association scores which quantify the association between genes hosting candidate variants and diseases of interest. Based on large-scale simulation studies, we conclude that our method has satisfactory performance and competitive accuracy over existing methods. It is expected that Glints can serve as a useful tool in human genetics studies based on exome sequencing, and it can save time and cost for follow-up experimental studies and facilitate discovery of disease-causing variations.

## Methods

### Multivariate linear regression for association inference

With the assumption that phenotype similarity between two diseases can be explained by genotype similarity between them, known as the “guilt by association” principle [63], Glints extends our previous work [55, 64] and simultaneously regresses three types of phenotype similarities derived from HPO, MeSH and UMLS by each of the nine functional similarities between genes. In detail, given two diseases indexed by *d* and *e*, we present their phenotype similarities as **y**_*de*_ = (*y*_1_, *y*_2_, …, *y*_*p*_)^*T*^ with *p* = 3. For a given gene functional similarity measure (e.g., gene expression), we define the genotype similarity between the two diseases as

1 $$ {x}_{de}={\displaystyle \sum_{g\in \mathbf{D}}}{\displaystyle \sum_{h\in \mathbf{E}}}{\varphi}_{gh} $$

where **D** and **E** represents sets of genes known as associated with diseases *d* and *e*, respectively and *φ*_*gh*_ functional similarity between two genes *g* and *h* under the given functional similarity measure. The multivariate regression model is then written as

2 $$ {\mathbf{y}}_{de}=\mathbf{a}+{x}_{de}\mathbf{b}+{\mathbf{e}}_{de} $$

with **a**, **b** the regression intercept and slope, respectively and **e**_*de*_ a *p* dimensional Gaussian noise.

In order to characterize the strength of association between a candidate gene *g* and a query disease *d*, we assume that *g* is the only gene associated with disease *d*, and we rewrite the regression model as

3 $$ \mathbf{Y}=\mathbf{X}\boldsymbol{\Theta } +\mathbf{E} $$

where **Y** = (**y**_1_, …, **y**_*p*_)_*n* × *p*_ with **y**_*i*_ = (*y*_1*i*_, …, *y*_*ni*_)^*T*^_*  n* × 1_ for *i* = 1, …, *p* denotes the *p* different types of phenotype similarities between disease *d* and all other *n* diseases, **X** = (**1**, **x**) with **1**_*n*_ = (1, …, 1)^*T*^_*  n* × 1_ and **x** = (*x*_1_, …, *x*_*n*_)^*T*^_*  n* × 1_ the corresponding genotype similarities between disease *d* and all other *n* diseases (in other words, similarities between gene *g* and all genes known as associated with other diseases), **Θ** = (**θ**_1_, …, **θ**_*p*_)_2 × *p*_with **θ**_*i*_ = (*α*_*i*_, *β*_*i*_)^*T*^, ***α*** = (*α*_1_, …, *α*_*p*_) and **β** = (*β*_1_, …, *β*_*p*_) the vector of regression intercepts and slopes for *p* different types of phenotype similarities, **E** = (**ε**_1_, …, **ε**_*n*_)^*T*^_*  n* × *p*_with **ε**_*i*_ = (*ε*_*i*1_, …, *ε*_*ip*_)^*T*^ and **ε**_*i*_ ∼ *N*(**0**, **Σ**_**ε**_) for *i* = 1, …, *n* iid Gaussian noise.

We solve this regression model through maximum likelihood estimation and obtain point estimators of the parameters *α*, *β* and **Σ**_**ε**_ as

4 $$ \widehat{\boldsymbol{\alpha}}=\overline{\mathbf{y}}-\overline{x}\widehat{\boldsymbol{\upbeta}} $$

5 $$ \widehat{\boldsymbol{\upbeta}}={\left({\mathbf{x}}_c^T{\mathbf{x}}_c\right)}^{-1}{\mathbf{x}}_c^T{\mathbf{Y}}_c $$

6 $$ {\widehat{\boldsymbol{\Sigma}}}_{\boldsymbol{\upvarepsilon}}={\left(n-2\right)}^{-1}{\widehat{\mathbf{E}}}^T\widehat{\mathbf{E}} $$

where ${\mathbf{x}}_c={{\left({x}_1-\overline{x},\dots, {x}_n-\overline{x}\right)}^T}_{n\times 1}$, ${\mathbf{Y}}_c={\left({\mathbf{y}}_1-{\mathbf{1}}_n\cdot {\overline{y}}_1,\dots, {\mathbf{y}}_p-{\mathbf{1}}_n\cdot {\overline{y}}_p\right)}_{n\times p}$ with $\overline{\mathbf{y}}={\left({\overline{y}}_1,\dots, {\overline{y}}_p\right)}_{1\times p}$ and $\widehat{\mathbf{E}}={\mathbf{Y}}_c-{\mathbf{x}}_c\widehat{\boldsymbol{\upbeta}}$. Furthermore, sampling distributions for these estimations are:

7 $$ \widehat{\boldsymbol{\upbeta}}\sim N\left(\boldsymbol{\upbeta}, {\left({\mathbf{x}}_c^T{\mathbf{x}}_c\right)}^{-1}{\boldsymbol{\Sigma}}_{\boldsymbol{\upvarepsilon}}\right) $$

8 $$ \left(n-2\right){\widehat{\boldsymbol{\Sigma}}}_{\boldsymbol{\upvarepsilon}}={\widehat{\mathbf{E}}}^T\widehat{\mathbf{E}}\sim {W}_p\left({\boldsymbol{\Sigma}}_{\boldsymbol{\upvarepsilon}},n-2\right) $$

where *W*_*p*_ stands for the Wishart distribution.

To infer whether the candidate gene is associated with the query disease, we test the relationship between the phenotype similarities and the genetic similarity, i.e., the capability of explaining the phenotype similarities using the genetic similarity. If target gene *g* is associated with query disease *d*, we should observe positive relationship. Since we have *β*_1_, …, *β*_*p*_ to represent correlations between genetic similarity and *p* different phenotype similarity, we seek for simplicity to test the hypothesis:

9 $$ {H}_0:{\beta}_1+\dots +{\beta}_p=0\kern0.7em vs\kern0.5em {H}_1:{\beta}_1+\dots +{\beta}_p>0 $$

Obviously, a significant *p*-value for rejecting the null hypothesis represents strong support against the correlation between the genetic similarity and the phenotype similarities and leads to strong evidence for association between the candidate gene and the query disease. A more general form of (9) is:

10 $$ {H}_0:{\boldsymbol{\uplambda}}^T\boldsymbol{\upbeta} =0\kern0.7em vs\kern0.5em {H}_1:{\boldsymbol{\uplambda}}^T\boldsymbol{\upbeta} >0 $$

where **λ** is the weight of different phenotype similarity measures and we set **λ** = (1, 1, 1)^*T*^ in our studies. To solve this problem, we propose a statistic that is subjected to the student *t* distribution (derivation in Additional file 1: Section 4), as:

11 $$ T=\frac{{\boldsymbol{\uplambda}}^T\widehat{\boldsymbol{\upbeta}}-{\boldsymbol{\uplambda}}^T\boldsymbol{\upbeta}}{{\left({\boldsymbol{\uplambda}}^T{\left({\mathbf{x}}_c^T{\mathbf{x}}_c\right)}^{-1}{\widehat{\boldsymbol{\Sigma}}}_{\boldsymbol{\upvarepsilon}}\boldsymbol{\uplambda} \right)}^{1/2}}\sim {t}_{n-2} $$

Therefore, by selecting the test statistic as

12 $$ T=\frac{{\boldsymbol{\uplambda}}^T\widehat{\boldsymbol{\upbeta}}}{{\left({\boldsymbol{\uplambda}}^T{\left({\mathbf{x}}_c^T{\mathbf{x}}_c\right)}^{-1}{\widehat{\boldsymbol{\Sigma}}}_{\boldsymbol{\upvarepsilon}}\boldsymbol{\uplambda} \right)}^{1/2}} $$

we calculate a *p*-value as *p* = *P*(*t*_*n* − 2_ ≥ *T*), which characterize statistical significance of association between candidate gene and query disease.

### Calibration of *p*-values

We used 14 types of functional prediction scores on variant-level and 9 types of association scores on gene-level. Those scores are heterogeneous for quantities and implications, which makes it difficult to integrate them directly. Therefore, before integration, we converted all those scores into *p*-values, which can be integrated with Fisher’s method.

For each type of variant-level score, we first sorted all available scores stored in a database (e.g. dbWGFP) and built an empirical null distribution after excluding known causal variants. Although some unknown causal variants may still exist, their impact on the estimation of the empirical null distribution is negligible, due to the low odds of causal to neutral variants, which results from natural selection. Then, we compared a query score with the corresponding empirical null distribution and calculated the proportion of more extreme scores as the empirical *p*-value. For SIFT and LRT, smaller scores indicate higher deleteriousness, and hence “more extreme” means smaller than the query score. For all the other scores, larger scores indicate higher deleteriousness, and “more extreme” means greater than the query score.

For gene-level scores, it is also necessary to calculate empirical *p*-value. Although we can obtain analytical *p*-values from regression analysis as detailed above, those *p*-values can be biased when the underlying assumption is violated. We therefore built an empirical null distribution for each type of association score from corresponding analytical *p*-values of neutral genes, which are not reported as causative for any diseases. Then, for each gene-level analytical *p*-value, we compared it with corresponding empirical null distribution and calculate the proportion of more extreme values as empirical *p*-values. Here “more extreme” means smaller than the analytical *p*-value of the query gene.

### Fisher’s method with dependence correction

We adopted a Fisher’s method to combine *p*-values obtained from different data sources. Specifically, given the *p*-values to be integrated, denoted as *p*_1_, …, *p*_*K*_, where *K* denotes the total number of different data sources. We defined the Fisher’s statistic as

13 $$ U={\displaystyle \sum_{i=1}^K}{V}_i $$

where *V*_*i*_ = − 2 log *p*_*i*_. It is evident that *p*_*i*_ ~ *Uniform*[0, 1] and *V*_*i*_ ~ *χ*_2_^2^ under the null hypothesis. Since obvious correlations exist between different data sources, we assume that *U* follows a scaled chi-squared distribution with scale *η* and degrees of freedom *v* under the null hypothesis. We then adopted the method of moments to derive the matching equations as

14 $$ \begin{array}{l}E\left[\eta {\chi}_v^2\right]=\eta v=E\left[\mathrm{U}\right]=2K\\ {}Var\left[\eta {\chi}_v^2\right]=2{\eta}^2v=Var\left[\mathrm{U}\right]=4{\displaystyle \sum_{i=1}^K{\displaystyle \sum_{j=1}^K}}\operatorname{cov}\left({V}_i,{V}_j\right)\end{array} $$

and obtain parameter estimates as

15 $$ \widehat{\eta}=\frac{{\displaystyle {\sum}_{i=1}^K{\displaystyle {\sum}_{j=1}^K\operatorname{cov}\left({V}_i,{V}_j\right)}}}{K^2}\kern0.55em \mathrm{and}\kern0.45em \widehat{v}=\frac{2}{\widehat{\eta}}K $$

We estimated cov(*V*_*i*_, *V*_*j*_) with the method proposed by Yang. We first convert a *p*-value *p*_*i*_ into a statistic *z*_*i*_ via normal transformation *z*_*i*_ = Φ^− 1^(1 − *p*_*i*_), where Φ is the cumulative function of the standard normal distribution, and it is obvious that *z*_*i*_ ~ *N*(0, 1) under the null hypothesis. As suggested in Yang [53], let

16 $$ {\widehat{\rho}}_{ij}=Cor\left({Z}_i,{Z}_j\right)\kern0.65em \mathrm{and}\kern0.8em {\tilde{\rho}}_{ij}={\widehat{\rho}}_{ij}\left(1+\frac{1-{\widehat{\rho}}_{ij}^2}{2n-1}\right) $$

We calculated the covariance as

17 $$ Cov\left({V}_i,{V}_j\right)={a}_1{\tilde{\rho}}_{ij}+{a}_2{\tilde{\rho}}_{ij}^2+{a}_3{\tilde{\rho}}_{ij}^3+{a}_4{\tilde{\rho}}_{ij}^4 $$

where *a*_1_ = 3.263119, *a*_2_ = 0.709866, *a*_3_ = 0.026589, *a*_4_ = − 0.709866/*n*, with *n* the sample size.

We also calculated q-values [54] for the combined *p*-values to control positive false discovery rate (pFDR), which showed significant improvement in power in some studies compared with the traditional Benjamini-Hochberg approach [65]. It is also desirable that our method can easily handle the missing data source problem, in which we decreased the total number of *p*-values to be combined.

## Additional file

Additional file 1: Detailed information about data preprocessing. (DOC 2331 kb)
