# Supplementary material for: Global inference of disease-causing single nucleotide variants from exome sequencing data
Source: BMC Bioinformatics. 2016 Dec 23;17(Suppl 17):468. doi: 10.1186/s12859-016-1325-x (PMC5260102; doi:10.1186/s12859-016-1325-x)
Supplement: Additional file 1: — Detailed information about data preprocessing. (DOC 2331 kb) [file 12859_2016_1325_MOESM1_ESM.doc]

# Supplementary Materials for Global inference of disease-causing single nucleotide variants from exome sequencing data

Mengmeng Wu^1,2^, , Ting Chen^1,2^ and Rui Jiang^1,3*^

^1^ MOE Key Laboratory of Bioinformatics; Bioinformatics Division and Center for Synthetic & Systems Biology, TNLIST

^2^ Department of Computer Science, Tsinghua University

^3^ Department of Automation, Tsinghua University

*To whom correspondence should be addressed.

## Text mining for phenotype similarities

We constructed three phenotype similarity matrices and disease network based on text mining technique, including HPO, MeSH and UMLS.

*HPO* We collected 10,436 HPO concepts and corresponding annotations for 6,346 human diseases . We characterized each disease with a 10,436 dimensional numeric vector, and each dimension is computed as the negative logarithm of the corresponding concept’s frequency, which represents the information about the concept. We also added the frequency of one concept to parent node recursively due to the structure (directed acyclic graph or DAG) of HPO ontology. For each pair of diseases, we then computed the cosine of the angle between their concept vectors as their similarity scores, which is proved to be a simple but effective measures. We obtained a matrix for measuring phenotype similarities between all those human diseases by applying this method to each pair of diseases, and we then kept only 10 nearest diseases for each disease to remove low confident edges. Therefore, we obtained a nearest neighbor network called HPO, and 10 is the optimal number of nearest neighbor according to literature.

*MeSH* We collected 7,719 disease texts from the OMIM database and break those texts into words by splitting sentences in the TX and the CS fields. We obtained 5,632 MeSH concepts by mapping these words from OMIM database onto MeSH concepts with the MetaMap program . We characterized each disease with a 5,632 dimensional numeric vector, and each dimension represents the frequency of corresponding concept. We then applied the aforementioned cosine measure to compute pairwise phenotype similarity, and obtained the final nearest neighbor network, called MeSH, with the above nearest neighbor strategy.

*UMLS* Similar to MeSH, we collected the same set of diseases and performed the same splitting procedure. We then mapped words onto UMLS concepts, instead of MeSH concepts. We repeated the same construction procedure, and obtained a nearest neighbor network, called UMLS.

## Construction of gene similarity networks

We constructed nine gene similarity networks from nine different genomic data, including gene expression (gexp), gene ontology (gobp), pathway membership (kegg), protein sequence (pseq), protein domain (pfam), protein-protein interaction (strg), transcriptional regulation (tsfc), microRNA regulation (mrna) and signaling regulation (sign).

***gexp*** We collected genome-wide assays of 44,775 transcripts across 79 tissues , and represented every gene as a 79-dimensional vector, each of which denoted the expression value in one tissue. We then calculated the absolute value of Pearson’s correlation coefficient between each pair of genes’ expression vectors as raw similarity between the two genes. Finally, we derived the gene expression similarity and filtering weak signals by applying an exponential transformation to the raw similarities, as

(1)

where $\omega_{gh}^{(\mathrm{gexp})}$ is the raw similarity between genes *g* and *h*, $\sigma^{(g\exp)}$ the standard deviation of all $\omega_{gh}^{(\mathrm{gexp})}$, and the gene expression similarity.

***gobp*** We collected 25,626 gene ontology terms from the biological process domain and represent each gene as a 25,626 dimensional vector, each of which denoted the negative logarithm of the frequency of the corresponding term in annotations. We then calculated the cosine of the angle between the corresponding vectors as the raw similarity between each pair of genes and applied the aforementioned exponential transformation to derive the gene ontology similarity matrix.

***kegg*** We collected 238 human pathways after removing disease-related ones to eliminate bias towards well-studied diseases from the KEGG database and represent each gene as a 238 dimensional binary vector, each of which denoted whether the gene is involved in the corresponding pathway. We then calculated cosine of angle between the corresponding vectors as raw similarity between each pair of genes and applied the exponential transformation to derive the gene pathway similarity matrix.

***pseq*** We collected 20,274 protein sequences from Swiss-Prot database and calculated pairwise similarity using Smith-Waterman algorithm implemented in SSEARCH. We constructed an undirected network and connected two proteins if the e-value for alignment between them was less than 10^-4^. We then calculated the shortest path distance between each pair of proteins on the network as $\delta_{gh}^{(\mathrm{pseq})}$(for protein g and h), and derive the raw similarity with a linear transformation, $\omega_{gh}^{(\mathrm{pseq})}=1-\delta_{gh}^{(\mathrm{pseq})}/\max\delta_{gh}^{(\mathrm{pseq})}$. Finally, we adopted the exponential transformation to derive the protein sequence similarity matrix.

***pfam*** We collected 14,831 protein domains from the Pfam database (Version 27.0) and represented each protein as a 14,831 dimensional binary vector, each of which denoted whether the protein existed in the corresponding domain. We calculated the cosine of angle between corresponding vectors as raw similarity for each pair of proteins and applied the aforementioned exponential transformation to derive final protein domain similarity matrix.

***strg*** We collected 403,514 interactions between 13,747 proteins from the STRING database (Version 9.1) and constructed an undirected protein-protein interaction network. We then calculated pairwise shortest path distances between proteins like the derivation of protein sequence similarity, and obtained raw similarity between each pair of genes by the linear transformation. Finally, we applied the exponential transformation to derive protein interaction similarity matrix.

***tsfc*** We collected 218 position weighted matrices (PWM) of vertebrate transcription factors with high confidence and identified potential binding sites in promoter regions (1,000 basepairs upstream) of each human gene with program MATCH. We then represented each gene as a 218-dimensional vector, each of which denoted the frequency of the promoter region of the gene bound by the corresponding transcription factor. We then calculate raw similarity between two genes using the cosine measure and derived the transcriptional regulation similarity according to the exponential transformation.

***mrna*** We collected 249 microRNAs from the miRanda database and represented a gene as a 249 dimensional binary vector, each of which denotes whether the gene is a target of a microRNA. We then calculate the cosine of angle between corresponding vectors of each pair of genes as the raw similarity and applied the aforementioned exponential transformation to derive the microRNA regulation similarity matrix.

***sign*** We extracted signaling interactions from a human signaling network, including 33,398 activation, 7,960 inhibition and 21,579 physical interaction. We constructed a hybrid network by assigning directed edges to activation or inhibition and undirected edges to physical interactions. We then computed the shortest path distance $\delta_{gh}^{(\mathrm{sign})}$ for every pair of proteins g and h and converted it into 0 to 1 with $\omega_{gh}^{(\mathrm{sign})}=1-\delta_{gh}^{(\mathrm{sign})}/\max\delta_{gh}^{(\mathrm{sign})}$. Finally, we applied the aforementioned exponential transformation to obtain the final signaling interaction similarity matrix.

## Derivation of statistic T

Here, we present the detailed derivation of sampling distribution of statistic T proposed in equation (11) in main text. As suggested by Johnson , we can see that:

It is also easy to obtain:

Then, we can derive that:

Next, we can derive that:

$$\lambda^{T}\hat{\beta}\sim N(\lambda^{T}\beta,\lambda^{T}\Sigma_{\beta}\lambda)$$

$$\frac{\lambda^{T}\hat{\beta}-\lambda^{T}\beta}{(\lambda^{T}\Sigma_{\beta}\lambda)^{1/2}}\sim N(0,1)$$

Thus, we can obtain the distribution of statistic T as:

$$T=\frac{\lambda^{T}\hat{\beta}-\lambda^{T}\beta}{(\lambda^{T}\hat{\Sigma}_{\beta}\lambda)^{1/2}}=\frac{\lambda^{T}\hat{\beta}-\lambda^{T}\beta}{(\lambda^{T}\Sigma_{\beta}\lambda)^{1/2}\left(\frac{(n-p)}{(n-p)}\frac{\lambda^{T}\hat{\Sigma}_{\beta}\lambda}{\lambda^{T}\Sigma_{\beta}\lambda}\right)^{1/2}}=\frac{N(0,1)}{\sqrt{\chi_{n-2}^{2}/(n-2)}}\sim t_{n-2}$$

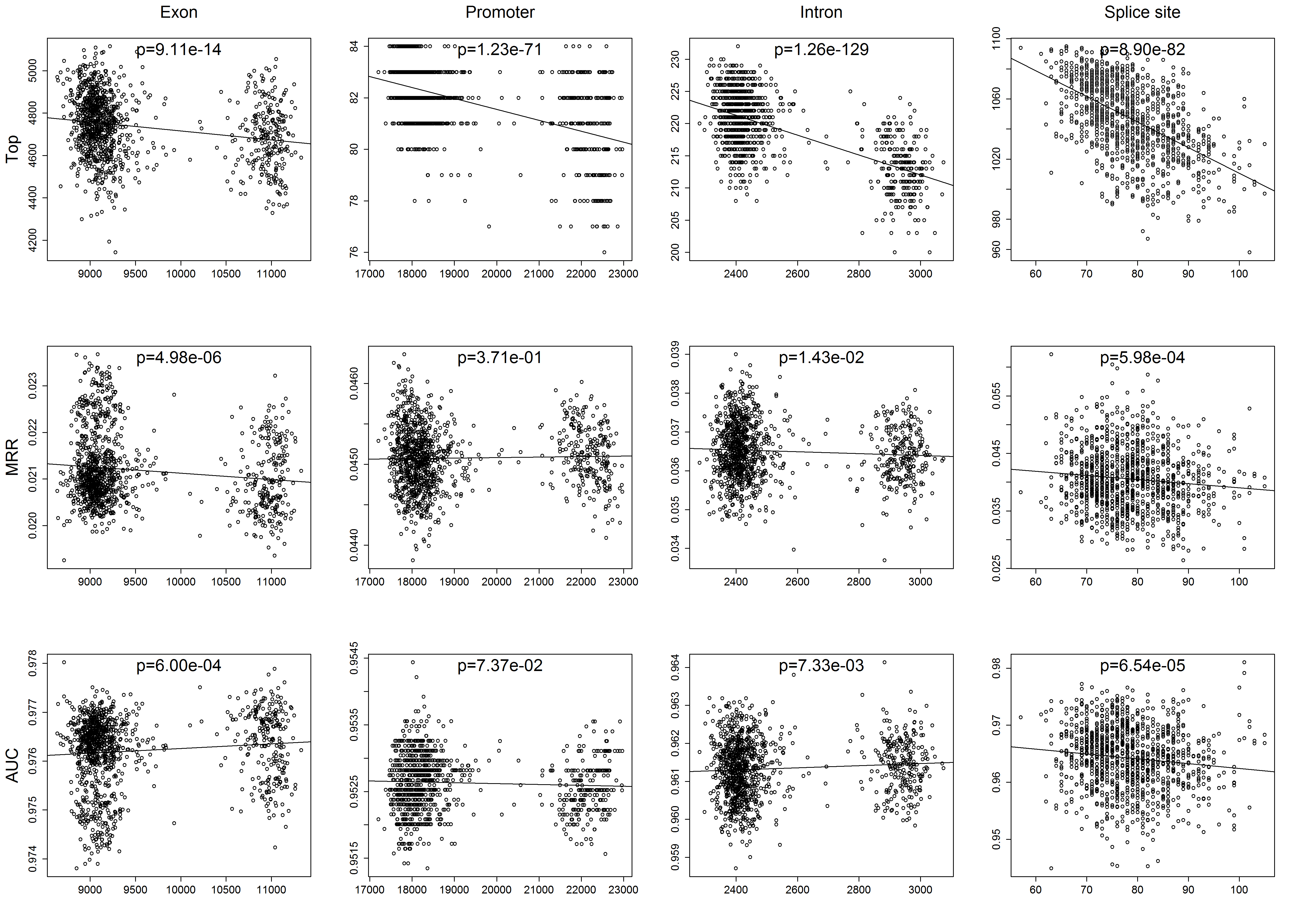
**Supplementary Figure 1. The influence of candidate number on prioritization performance.** The four colums correspond to different regions, as Exon, Promoter, Intron and Splice site. The three rows correspond to three different metrics, as Top, MRR and AUC.


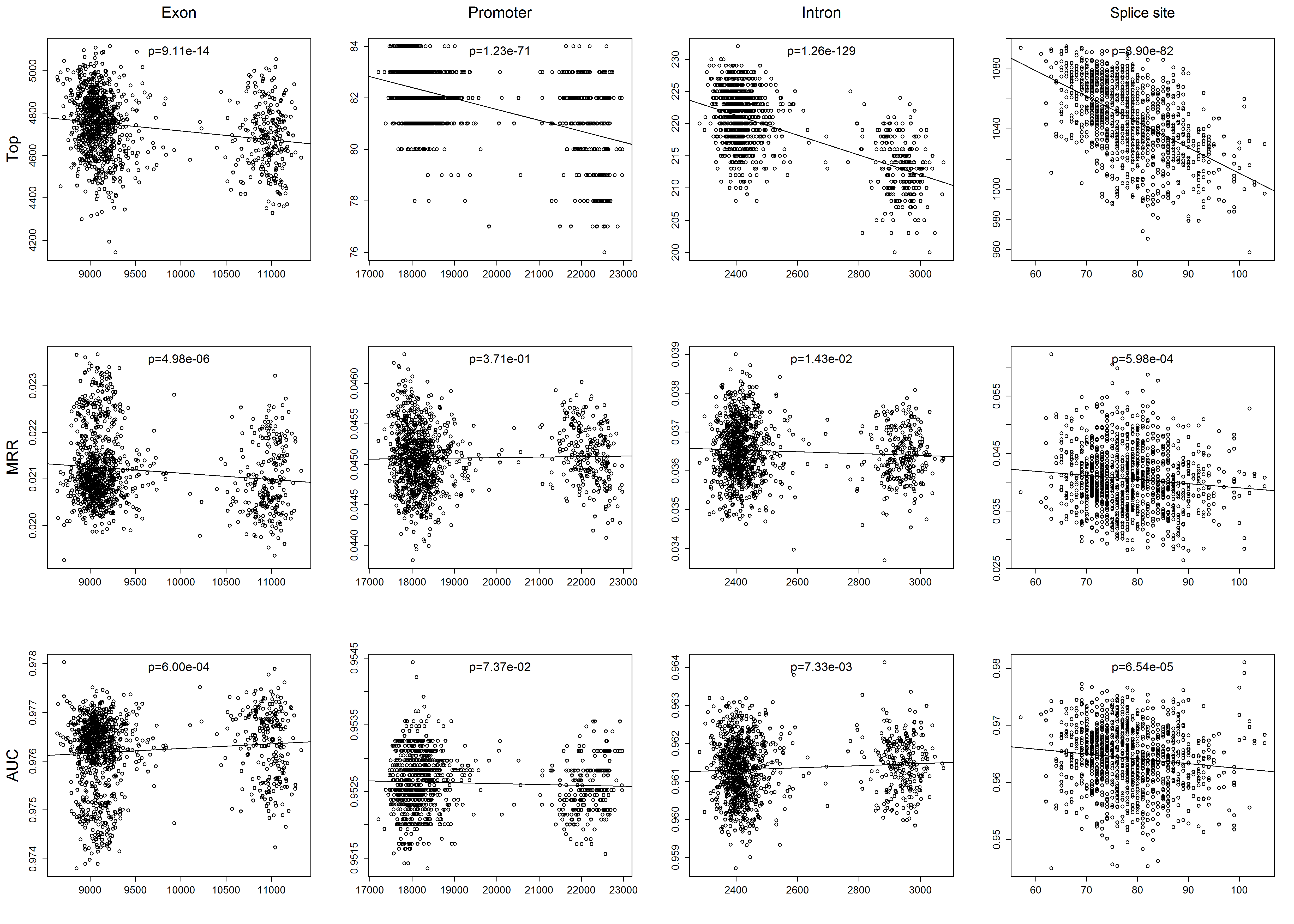

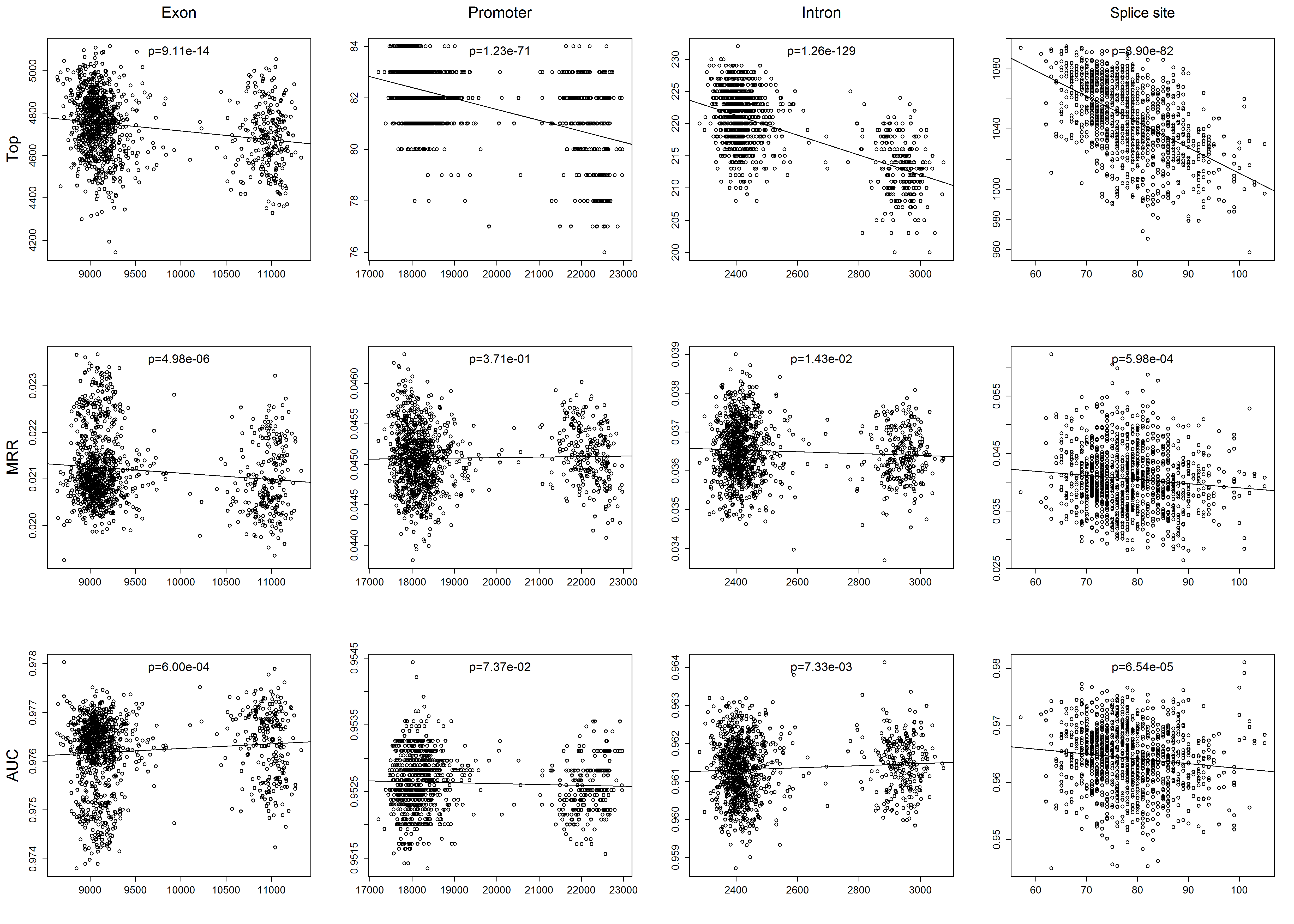


**Supplementary Table 1. coverage of functional and association scores.** Neutral variants refer to the pools of all KG neutral variants across different regions. Some abbreviations for score name: gexp, gene expression; gobp, gene ontology; kegg, KEGG pathway; mrna, microRNA regulation; pfam, protein families; pseq, protein sequence; sign, signaling pathway; strg, protein-protein interaction; tsfc, transcriptional regulation;

| **Name** | **Exon** | | **Promoter** | | **Intron** | | **Splice site** | |
| --- | --- | --- | --- | --- | --- | --- | --- | --- |
|  | Disease | neutral | disease | Neutral | Disease | Neutral | Disease | neutral |
| CADD | 100% | 99.0% | 100% | 97.5% | 100% | 100% | 100% | 100% |
| DANN | 100% | 99.0% | 100% | 97.5% | 100% | 100% | 100% | 100% |
| FATHMM-MKL | 100% | 99.0% | 100% | 97.5% | 100% | 100% | 100% | 100% |
| Eigen | 100% | 97.1% | 72.8% | 95.4% | 99.0% | 96.0% | 100% | 98.1% |
| LRT | 96.4% | 51.1% | 0.0% | 0.0% | 0.2% | 0.1% | 0.1% | 0.3% |
| MSRV | 99.4% | 53.1% | 0.0% | 0.0% | 0.0% | 0.0% | 0.0% | 0.1% |
| MutationAccessor | 99.0% | 55.9% | 0.0% | 0.0% | 0.0% | 0.0% | 0.0% | 0.1% |
| PolyPhen2 | 100% | 56.7% | 0.0% | 0.0% | 0.0% | 0.3% | 0.2% | 0.7% |
| SinBaD | 100% | 61.3% | 0.0% | 0.1% | 0.6% | 1% | 99.3% | 85.8% |
| SIFT | 96.1% | 56.6% | 0.0% | 0.1% | 0.2% | 0.5% | 4.8% | 0.9% |
| GERP | 100% | 99.0% | 100% | 97.5% | 100% | 100% | 100% | 100% |
| Siphy | 99.9% | 99.0% | 100% | 96.7% | 99.8% | 99.9% | 99.9% | 99.9% |
| Phylop | 100% | 99.0% | 100% | 97.3% | 100% | 99.9% | 100% | 99.9% |
| PhastCons | 100% | 99.0% | 100% | 97.3% | 100% | 99.9% | 100% | 99.9% |
| gexp | 87.1% | 66.6% | 89.7% | 70.4% | 80.2% | 74.8% | 89.6% | 87.6% |
| gobp | 92.8% | 82.7% | 92.3% | 85.0% | 90.3% | 86.7% | 90.4% | 91.6% |
| kegg | 43.6% | 34.5% | 64.1% | 36.1% | 46.0% | 37.2% | 46.3% | 45.2% |
| mrna | 75.7% | 71.0% | 87.2% | 73.5% | 76.6% | 74.6% | 80.1% | 77.2% |
| pfam | 62.5% | 59.5% | 71.8% | 61.6% | 65.3% | 61.3% | 68.2% | 65.2% |
| pseq | 60.5% | 52.5% | 53.8% | 53.6% | 62.0% | 53.4% | 60.0% | 58.6% |
| sign | 38.4% | 26.8% | 43.6% | 28.6% | 36.7% | 30.4% | 38.4% | 37.0% |
| strg | 78.2% | 64.8% | 79.5% | 67.8% | 75.6% | 70.4% | 79.6% | 78.9% |
| tsfc | 100% | 99.9% | 100% | 100% | 100% | 100% | 100% | 100% |

**Supplementary Table 2. Training datasets and methods used in each functional scores.**

| Scores | Positive training data | Negative training data | Training method |
| --- | --- | --- | --- |
| CADD | De novo mutation simulated with GTR model | MAF > 95% in 1000 Genomes Project (20110521, Phase I) and match ancestral sequence | SVM |
| DANN | Same as above | Same as above | Neural Network |
| FATHMM-MKL | HGMD | MAF > 1% in 1000 Genomes Project (Phase I) | Multiple Kernel Learning |
| LRT | Multiple sequence alignment of protein-coding sequences of 32 vertebrate species | | Likelihood ratio test |
| MSRV | Swiss-prot | Swiss-prot | Multiple selection rule voting |
| MutationAccessor | Multiple sequence alignment of 700 homologous sequences from Uniprot | | Entropy model |
| PolyPhen2 | HumDiv, HumVar | HumDiv, HumVar | Naïve bayes |
| SinBaD | HGMD | Simulated variants | Logistic regression |
| SIFT | Multiple protein sequence alignment | | Estimate alignment probability |
| GERP, Siphy,  Phylop, PhastCons | Multiple sequence alignment | | Evolutionary model |

**References**

1. Robinson PN, Köhler S, Bauer S, Seelow D, Horn D, Mundlos S: **The Human Phenotype Ontology: a tool for annotating and analyzing human hereditary disease**. *The American Journal of Human Genetics* 2008, **83**(5):610-615.

2. Gan M: **Correlating information contents of gene ontology terms to infer semantic similarity of gene products**. *Computational and mathematical methods in medicine* 2014, **2014**.

3. Jiang R, Gan M, He P: **Constructing a gene semantic similarity network for the inference of disease genes**. *BMC systems biology* 2011, **5**(Suppl 2):S2.

4. Aronson AR: **Effective mapping of biomedical text to the UMLS Metathesaurus: the MetaMap program**. In: *Proceedings of the AMIA Symposium: 2001*. American Medical Informatics Association: 17.

5. Su AI, Wiltshire T, Batalov S, Lapp H, Ching KA, Block D, Zhang J, Soden R, Hayakawa M, Kreiman G: **A gene atlas of the mouse and human protein-encoding transcriptomes**. *Proceedings of the National Academy of Sciences of the United States of America* 2004, **101**(16):6062-6067.

6. Ashburner M, Ball CA, Blake JA, Botstein D, Butler H, Cherry JM, Davis AP, Dolinski K, Dwight SS, Eppig JT: **Gene Ontology: tool for the unification of biology**. *Nature genetics* 2000, **25**(1):25-29.

7. Kanehisa M, Goto S: **KEGG: kyoto encyclopedia of genes and genomes**. *Nucleic acids research* 2000, **28**(1):27-30.

8. UniProt C: **The Universal Protein Resource (UniProt) in 2010**. *Nucleic acids research* 2010, **38**(Database issue):D142-148.

9. Li W, McWilliam H, Goujon M, Cowley A, Lopez R, Pearson WR: **PSI-Search: iterative HOE-reduced profile SSEARCH searching**. *Bioinformatics* 2012, **28**(12):1650-1651.

10. Bateman A, Coin L, Durbin R, Finn RD, Hollich V, Griffiths‐Jones S, Khanna A, Marshall M, Moxon S, Sonnhammer EL: **The Pfam protein families database**. *Nucleic acids research* 2004, **32**(suppl 1):D138-D141.

11. Snel B, Lehmann G, Bork P, Huynen MA: **STRING: a web-server to retrieve and display the repeatedly occurring neighbourhood of a gene**. *Nucleic acids research* 2000, **28**(18):3442-3444.

12. Matys V, Fricke E, Geffers R, Gößling E, Haubrock M, Hehl R, Hornischer K, Karas D, Kel AE, Kel-Margoulis OV: **TRANSFAC®: transcriptional regulation, from patterns to profiles**. *Nucleic acids research* 2003, **31**(1):374-378.

13. Cui Q, Ma Y, Jaramillo M, Bari H, Awan A, Yang S, Zhang S, Liu L, Lu M, O'Connor‐McCourt M: **A map of human cancer signaling**. *Molecular systems biology* 2007, **3**(1):152.

14. Johnson RA, Wichern DW: **Applied multivariate statistical analysis**, vol. 4: Prentice hall Englewood Cliffs, NJ; 1992.
